# An improved automatic liquid injection apparatus for use on a carbon analyser

**DOI:** 10.1155/S1463924679000274

**Published:** 1979

**Authors:** R. A. Van Steenderen

**Affiliations:** National Institute for Water Research of the Council for Scientific and Industrial Research P.O. Box 395 Pretoria 0001 South Africa


### An improved automatic liquid injection apparatus for use on a carbon analyser

**R. A. Van Steenderen**
*National Institute for Water Research of the Council for Scientific and Industrial Research, P.O. Box 395, Pretoria, 0001, Republic of South Africa.*

### Introduction

Water environmental studies necessitate the analytical processing of large numbers of samples for which automated techniques are an obvious requirement. In a previous article a fully automatic sample injection apparatus, for use in determining both inorganic and organic carbon on a Beckman carbon analyser, was described [1]. Sample volumes of 0.02 $cm^3$ were injected at a rate of 20/h. A modified injector which is considerably less complicated in structure and has fewer moving components, boosting the reliability of the operation, has now been developed. Furthermore, the instrument is (a) less bulky, i.e. both inorganic and total carbon injection units can be mounted on the same analyser unit; (b) less subject to sample carry-over since slide movement of the injector has been reduced to less than half that of the previous model; and (c) capable of handling four times the volume of the previous model, thereby considerably increasing instrument sensitivity. The latter is of particular importance since many laboratories are concerned with lower levels of TOC measurement than usually found in municipal or industrial wastes, e.g. reclamation processes or other sources of portable water supplies. The automated TOC analyser is non-specific but it can serve as a basic monitoring or screening mechanism for recording concentration variations or for subsequent more detailed analysis.

### Experimental

### Carbon analyser

Analysers used were:
(i) a Beckman 915 total organic carbon analyser (TOC analyser)
(ii) a Beckman Model 865 infra red detector employing 340 mm absorption cells for total carbon determination, and
(iii) a Beckman Model 215, an infra red analyzer employing 135 mm cells for inorganic carbon determination.

The carrier gas was oxygen at a flow rate of 2,5 $cm^3/s$ whilst the sample volumes injected were 0,08 and 0,03 $cm^3$ for total carbon and inorganic carbon respectively. The sampling rate was 20/h.

Processing a large number of consecutive samples using the above volumes required more efficient sample condensate removal than when employing smaller volumes. Water vapour entering the infra red analyser was kept to a minimum since the latter strongly absorbs throughout the infra red spectral region causing great disturbance to $CO_2$ measurements. Furthermore, inefficient vapour removal continually blocked the 'Hoke' filter with subsequent condensed water reaching the infra red analyser. For this purpose a glass U-tube was jacketed and cooled to 5 °C in order to remove the condensate before it passed into the infra red analyser. Recorder sensitivity was pre-set to 100 and 10 mV for total and inorganic carbon respectively and sensitivity adjustments were made to the detectors only.

### Technical details of the injection unit

The injection units were mounted on top of the carbon analyser as illustrated in Figure 1. The remaining manifold and components, described elsewhere [1], remained unaltered. Figure 2 illustrates the different parts used in the construction of the injection unit. Each component in Figure 2 is numbered and identified in Table 1.

### Operation of the injection unit

The operation of the injection unit is explained by Figure 2. The automatic sampler and the injection units are synchronised by means of a simple electronic timer. The automatic sampler takes samples of washwater and sample alternatively by being activated from position A to position B (a distance of 9 mm) and back at intervals of 90 seconds for a duration of 0,5 seconds. Position A is the resting position and liquid entering at point 7 via a

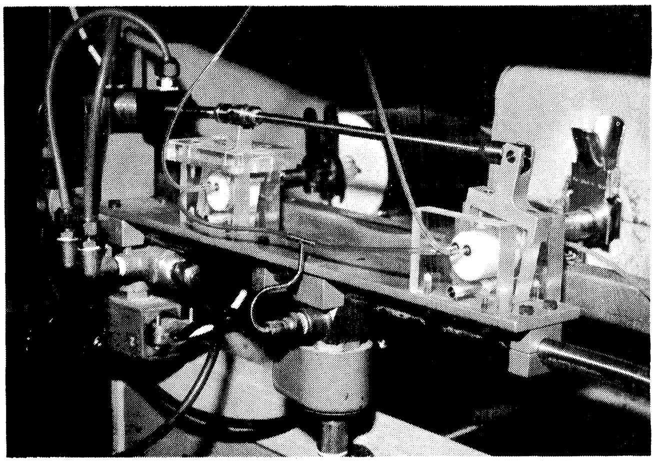

*Figure 1 Injection units affixed to the Beckman total organic carbon analyser.*

**Table I Parts list of the injection unit**

| Ref. No. | Component |
|---|---|
| 1. | Electrical power from sampler to solenoids 8. |
| 2. | Oxygen inlet to solenoid regulating the pressure for sample transfer to the combustion furnace. |
| 3. | Oxygen inlet to solenoid operating the pneumatic piston 4. |
| 4. | Pneumatic-operated piston device for driving the injectors. |
| 5. | Injection units. |
| 6. | Pressure inlet to combustion tube. |
| 7. | Sample carrier capillary. |
| 8. | Solenoid valves. |
| 9. | Connecting rod for simultaneous operation of the two injection units. |
| 10. | Stainless steel syringe needle of gauge 20 and length 90 mm. |
| 11. | Perspex walls 60 x 45 x 12 mm. Two rubber rings serve as seals in each wall. |
| 12. | A cylindrical Teflon disc which can be varied according to the volume required. |
| 13. | Pressure feed from solenoid to connection 6. |

**Table II Reproducibility using standard solutions of potassium biphthalate (Ten replicates)**

| N = 10 | Concentration/mg/dm³ | | | | |
|---|---|---|---|---|---|
| | 10 | 20 | 30 | 40 | 50 |
| x | 20,1 | 38,0 | 54,1 | 68,9 | 82,5 |
| SD | 0,5 | 0,6 | 0,5 | 0,7 | 0,9 |
| Cv | 2,8 | 1,7 | 1,0 | 1,0 | 1,1 |

Where: x = mean of recorded chart peak heights.
SD = Standard deviation.
Cv = Coefficient of variation.
N = Number of determinations.

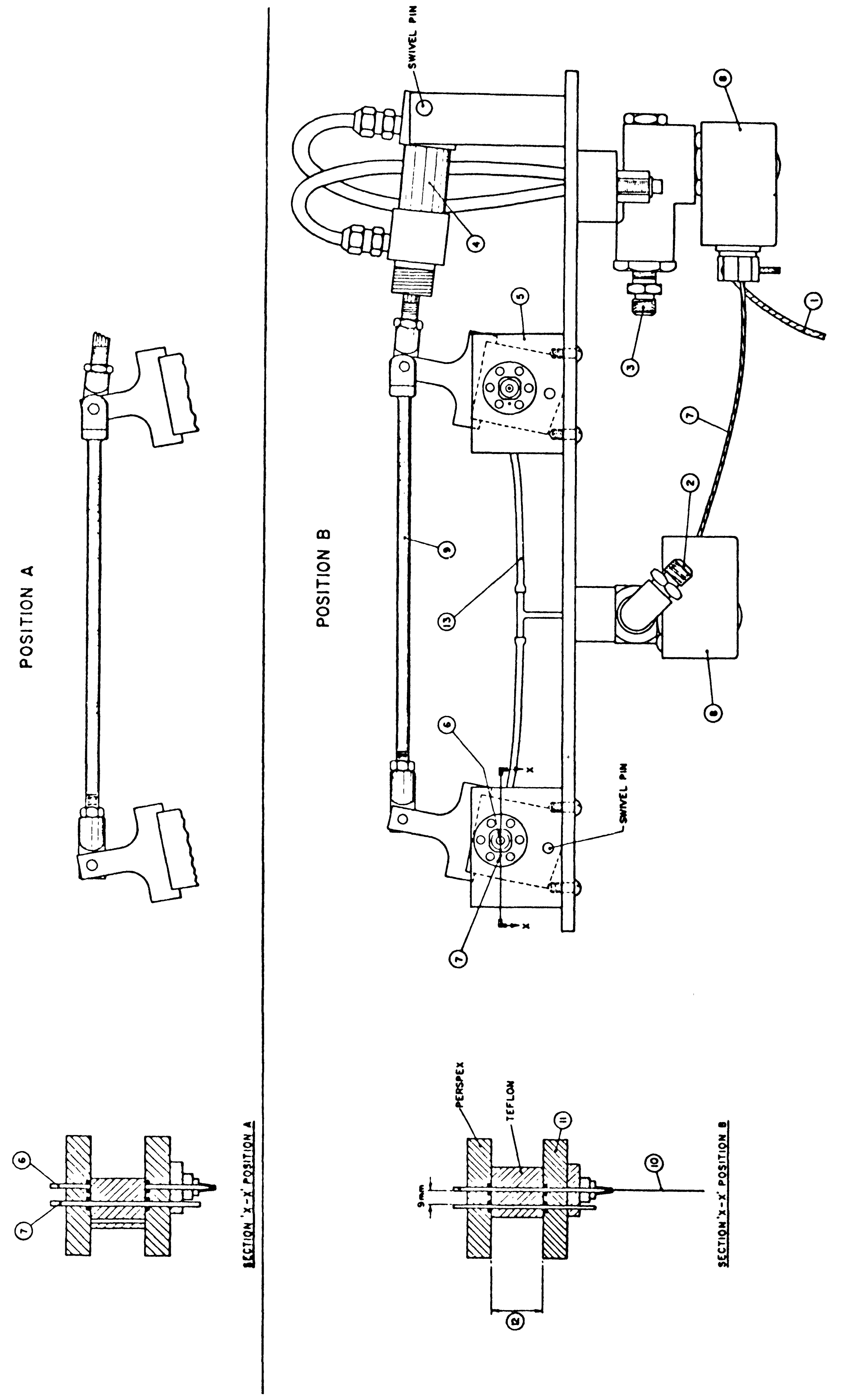

*Figure 2 Schematic diagram of sample injection unit.*

proportioning pump flows to waste, thereby ensuring that the capillary through the cylindrical Teflon block is always filled with liquid. Position B is obtained upon activation of the solenoid supplying oxygen at 50 kPa to the pneumatic piston 4. Simultaneously, oxygen at 60 kPa is supplied via connection 13 to inlet 6 of the injection unit where the capillary line containing the liquid collected while in position A is brought into alignment with the inlet port of the combustion furnace and driven into the furnace.

Oxygen is needed for the combustion stage and is used here to drive the injection assembly, any other gas would be equally suitable.

### Results and discussion

Since both injection units operated equally efficiently only the one connected to the total combustion furnace of the Beckman TOC analyser is discussed. The reproducibility of values obtained by using the injection unit were determined with standard solutions of potassium biphthalate for the range 10 to 50 mg/dm$^3$ C (Table 2).

Typical recorder tracings for a series of samples and standard solutions (Figure 3) indicate the consistent baseline that can be maintained. The recurring smaller peaks following each standard or sample peak represent washwater injections. These washwater peaks indicate the degree of contamination in the injection needle resulting from the previous injection. The irregular spikes in the baseline are caused by electronic interferences upon activation of the automatic sampler.

Automated TOC analysis for the range 0-10 mg/dm$^3$ using the described apparatus is also possible. (See Figure 4). It can be seen, however, that, in order to free the system from contamination, each sampling must be followed by two water washes, thereby reducing the sampling rate to 10/h. At this rate, therefore, automation may not be warranted.

An advantage of the injection units is that they can be constructed in any technical workshop. Compared with the first design [1] the injection units described in this article operate with much less mechanical movement and as a result are less subject to contamination carry-over from one sample to the next. Even with the low operating pressure of 50 kPa the slide mechanisms of the injectors were never inclined to stick after inoperative periods as

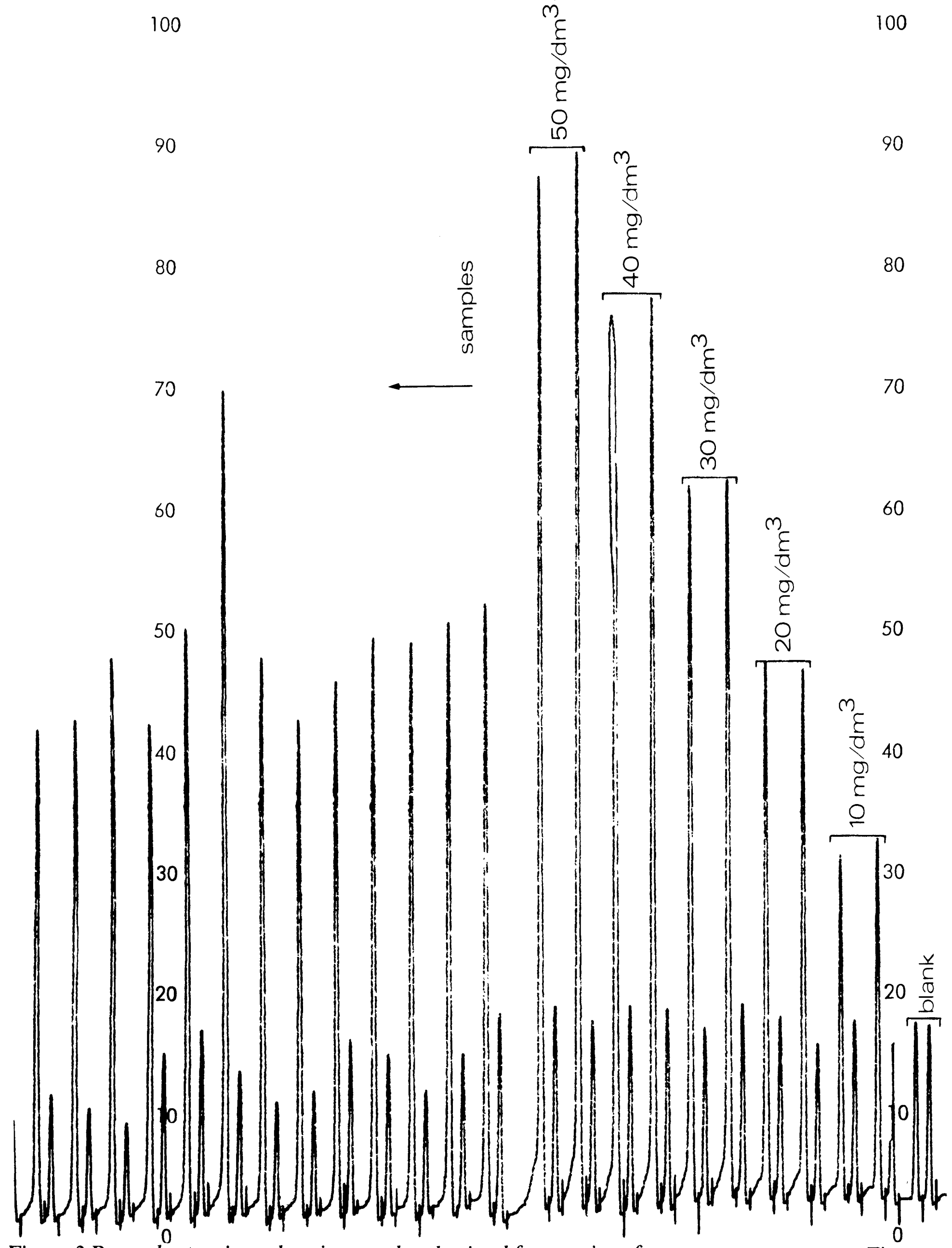

*Figure 3 Recorder tracings showing results obtained for a series of standards and samples.*

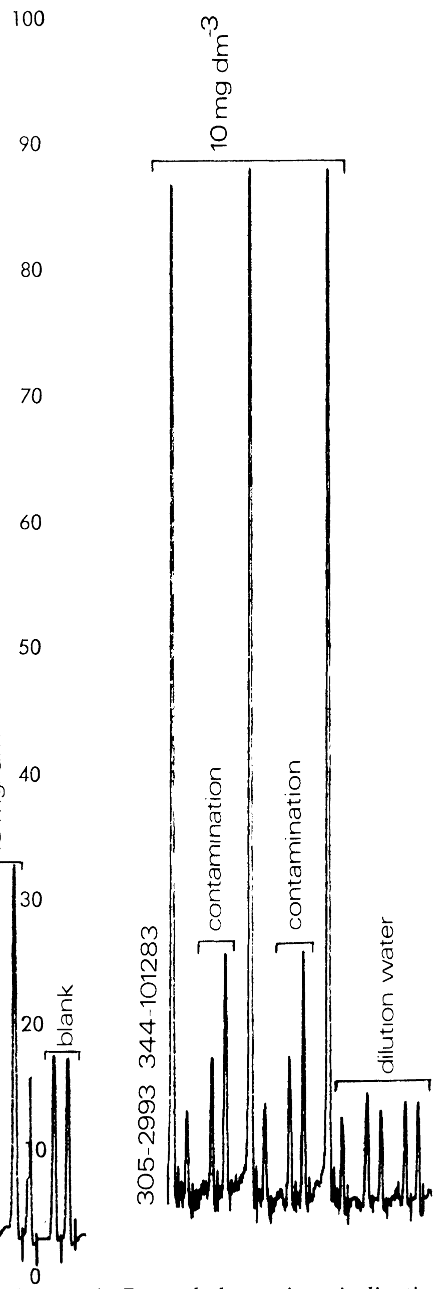

*Figure 4 Recorded tracing indicating the influence of contamination.*

was the case with the first design. Another advantage of the procedure described is that it enables the determination of total carbon and inorganic carbon in the range 10 to 50 $mg/dm^3$ at a rate of 20 samples/h at a standard deviation of less than 1 per cent.

The described automated system is used very effectively in the authors laboratory for routine sample analysis.

ACKNOWLEDGEMENTS
This work will form part of a D.Sc. thesis in the Faculty of Mathematics and Pure Sciences at the University of Pretoria.

A special word of thanks is extended to Mr. P. J. Meaton of the Technical Services Department for original suggestions and construction of the injection units.

This paper is published with the approval of the Director of the National Institute for Water Research.

REFERENCE

[1] Van Steenderen, R. A. (1976), *Water S.A.*, **2**, 126.

### An automated distillation method for the determination of diacetyl in beer: a comparison of analysis by AutoAnalyzer and gas chromatography

**J. C. Buijten* and B. Holm**
*The Department of Analytical Chemistry, Stockholm University, Stockholm, Sweden.*

A number of colorimetric methods for the determination of diacetyl are available in which diacetyl is converted to the nickel-salt of dimethylglyoxime, West et al [1], Kielhöfer et al [2], Canales et al [3] and Brenner et al [4].

Owades et al [5] developed a micro method for the colorimetric analysis of diacetyl in beer in which diacetyl was converted to the ferro-salt of dimethylglyoxime. This method has been modified by Drews et al [6].

Another colorimetric method, developed by Gjertsen et al [7], reacts diacetyl with o-phenylenediamine to produce, 2,3-dimethylchinaxolin which absorbs at 335 nm. All the above mentioned methods are based on distillation of the beer sample.

Harrison et al [8], Arkim [9] and Bärwald [10] developed gas chromatographic methods for the determination of diacetyl in beer using a head space technique and an electron-capture detector.

The aim of the work described in this paper was to develop a fully automated method for the colorimetric determination of diacetyl in beer using a Technicon AutoAnalyzer II system, coupled to a continuous flow distillation unit. This distillation technique had previously been applied to the determination of ethanol in blood, Buijten (11).

The results from the proposed method were compared statistically with the results from parallel determinations using the gas chromatographic method.

### Methods

### Distillation unit**

The all glass distillation unit according to Buijten [11], see Figure 1, has the following dimensions:

Coil 'H': inner diameter 15 mm, length 40 cm, coil height 'I' 15 cm, coil diameter 'J' 12 cm.

Tubes 'A', 'C', 'E' and 'F': outer diameter 4 mm, inner diameter 1.7 mm. Tube 'K': outer diameter 4 mm and inner diameter 1 mm.

* Department of Alcohol and Drug Addiction Research, Karolinska Institutet, S-104 01 Stockholm, Sweden.

**Swedish Patent No. 7300722—1.

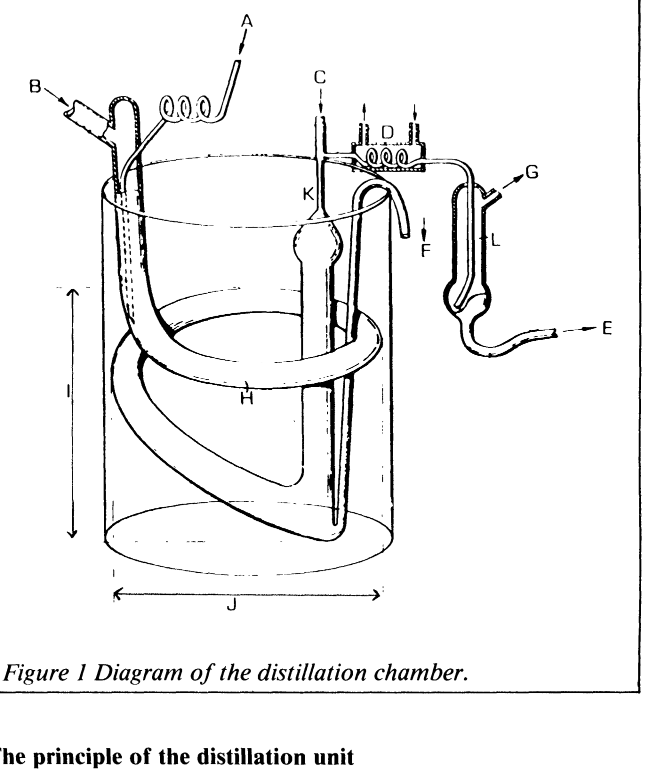

*Figure 1 Diagram of the distillation chamber.*

### The principle of the distillation unit

Volatile substances are separated from the sample by distillation in a distillation unit, which is coupled directly to a Technicon AutoAnalyzer. The samples are pumped consecutively into the distillation unit at 'A', Figure 1 and flow down into the warmed coil 'H'. Air, at approximately 2 litres per minute, is pumped in at 'B' and sweeps volatile components of the sample through to the cooling coil 'D' where they react with an absorbing reagent that has been pumped in at 'C'. The sample residue is pumped out at 'F', and excess air leaves the distillation unit at 'G'.

The absorbing reagent is pumped off at 'E' for further colorimetric analysis.

